# A comparative study of structural, functional and circulatory parameters in glaucoma diagnostics

**DOI:** 10.1371/journal.pone.0201599

**Published:** 2018-08-23

**Authors:** Natalia Ivanovna Kurysheva, Ekaterina Vladimirovna Maslova, Inna Vladimirovna Zolnikova, Alexey Valentinovich Fomin, Mikhail Borisovich Lagutin

**Affiliations:** 1 Consultative-Diagnostic Department of Ophthalmology Center of the Federal Medical and Biological Agency of the Russian Federation, A.I. Burnazyan Federal Medical and Biophysical Center of FMBA, Ophthalmological Department of the Institute of Improvement of Professional Skill of FMBA, Moscow, Russian Federation; 2 Moscow Helmholtz Research Institute of Eye Diseases, Moscow, Russian Federation; 3 National Research Institute of Eye Diseases, Russian Academy of Medical Sciences, Moscow, Russian Federation; 4 Lomonosov Moscow State University, Faculty of Mechanics and Mathematics, Department of Mathematical Statistics and Stochastic Processes, Moscow, Russian Federation; Universidade Federal do Rio de Janeiro, BRAZIL

## Abstract

**Purpose:**

To compare the diagnostic accuracy of structural parameters, vessel density (VD) measured by optical coherence tomography angiography (OCTA), and electrophysiological testing in diagnosis of primary open-angle glaucoma (POAG).

**Methods:**

35 healthy participants and 90 POAG subjects underwent the measurement of whole image en face (wi) VD in the disc/peripapillary region and macula, the retinal nerve fiber layer (RNFL), and the average thickness of ganglion cell complex (GCC), pattern electroretinograms and pattern visual evoked potentials. The area under the receiver operating characteristic curve (AUC) was assessed for each parameter to differentiate early POAG from healthy eyes and between the POAG stages.

**Results:**

To distinguish early POAG from healthy eyes, the parameters with the highest AUC were detected: P50 amplitude of transient pattern electroretinogram, 1° (AUC 0.93, p = 0.002), P1 component of steady-state pattern electroretinogram (AUC 0.92, p = 0.003), P100 amplitude of pattern visual evoked potential, 1° (AUC 0.84, p = 0.013), wiVD macula superficial (AUC 0.80, p = 0.001), wiVD Disc (AUC 0.74, p = 0.016), GCC (AUC 0.74, p = 0.016) and to distinguish early POAG from the moderate to severe POAG: inferotemporal peripapillary VD (AUC 0.94, p < 0.0001) and focal loss volume of GCC (AUC 0.92, p < 0, 001).

**Conclusions:**

Our results demonstrate the importance of measuring the microcirculation parameters in the macular area along with PERGs and PVEPs for the early detection of glaucoma. VD in the inferotemporal sector of the peripapillary retina and focal loss volume of the GCC are important for monitoring of the disease. The inclusion of OCTA, PERGs and PVEPs in glaucoma diagnostics may improve its early detection and monitoring.

## Introduction

For several decades, there has been a debate on which parameters–structural or functional–are of the highest diagnostic value in glaucoma [1]. For a long time perimetry remained the “gold standard” for diagnosis of primary open-angle glaucoma (POAG). According to the literature, the peripapillary retinal nerve fiber layer and the macular ganglion cell layer appear the most attractive structural markers for glaucoma diagnosis [2]. Moreover, some authors emphasized that the macular parameters had high discriminating power and high reproducibility for early glaucoma detection in comparison with the peripapillary retinal nerve fiber layer (RNFL) parameters [3].

Our recent studies have shown that the circulatory parameters can serve as the diagnostic markers of glaucoma [4]. Reduced retinal hemoperfusion in glaucoma has been repeatedly mentioned in the literature [5,6]. At present, there is much evidence suggesting hemodynamic impairments in the optic nerve head (ONH), retina, and retrobulbar circulation in glaucoma eyes [7–10]. Furthermore, some authors have also found that color Doppler imaging may have the predictive value for the visual function damage in glaucoma subjects [11,12].

However, it is not clear yet if reduced blood flow is the cause or the consequence of glaucoma damage secondary to retinal ganglion cell (RGC) death.

Optical coherence tomography (OCT) with angiography (OCTA) has opened the prospects for novel imaging of retinal and ONH microcirculation [13]. The method is based on a new 3-dimensional angiography algorithm called split-spectrum amplitude-decorrelation angiography (SSADA), which compares consecutive B-scans at the same location to detect flow using motion contrast. The reproducibility of the method has been reported in several studies [14–16].

Reduced ONH and peripapillary perfusion parameters have been reported by different authors in subjects with glaucoma measured by OCTA [13–18]. The decreased vessel density (VD) was significantly associated with the severity of visual field damage independent of the structural loss [19, 20] and diagnostic accuracy of its measurement were comparable to RNFL measurements in both POAG [20] and primary angle closure glaucoma (PACG) [21].

Previously, we have reported better diagnostic accuracy by using capillary density in the macular area over the peripapillary area and the optic disc in the early glaucoma detection [18]. These findings are consistent with the literature on early involvement of macula with its high concentration of RGCs in glaucoma and explain the localization of the vulnerable zone of retina affected at the very beginning of glaucoma [22,23].

The functional activity of RGCs can be most accurately measured via pattern electroretinogram (PERG). The pattern visual evoked potential (PVEP) is also an objective method for checking visual function. The changes in PERG and PVEP in glaucoma have been reported before the appearance of abnormalities in ONH and peripapillary retina [23–29]. Furthermore, PVEPs have been used to evaluate reversible ganglion cell damage in trials of neuroprotective agents for the treatment of glaucoma [30,31].

In this study, we hypothesize that the function of RGCs and their axons, as studied by electrophysiological methods and microcirculation parameters, may have high diagnostic accuracy along with structural changes in the glaucoma process.

The purpose of the research was to compare the diagnostic accuracy of structural parameters, VD measured by OCTA, and electrophysiological testing in the diagnosis of POAG.

## Methods

### Experimental design

The study was approved by the Ethical Committee (Institutional Review Board) of the Institution of Federal Medical and Biological Agency of Russia and was conducted in accordance with Good Clinical Practice within the tenets of the Declaration of Helsinki. Each subject was required to sign an informed consent form before being enrolled in the study and prior to any measurements being taken.

### Study subjects

One hundred twenty-five eyes of 125 subjects (90 subjects with glaucoma and 35 age-matched normal subjects) were included in this study. All subjects were northern Europeans.

POAG was diagnosed on the basis of characteristic changes in the optic disc detected by ophthalmoscopy, which was performed by one glaucoma specialist (NK) and confirmed by two other glaucoma specialists. These characteristic changes included pathological deviation from the normal neuroretinal rim, glaucomatous optic disc cupping, peripapillary atrophy, wedge-shaped defects of RNFL adjacent to the edge of optic disc, and hemorrhages on the optic disc boundary.

Eyes with POAG were divided according to the Hodapp-Parrish-Anderson grading scale of visual fields defect severity, which was determined using a Statpac-2 pattern deviation probability map of the 24–2, SITA-STANDARD visual field testing protocol, into groups: 1) early stage POAG group, which included forty-eight eyes with a mean deviation (MD) > −6 dB, less than 25% of points depressed below 5%, with less than 10 points depressed on the 1% level on pattern deviation, and all points in the central 5^0^ had a sensitivity of 15 dB); 2) moderate to severe stage POAG group contained forty-two eyes. The moderate to severe stage group included eyes with moderate stage (MD>−12 dB, less than 50% of the points depressed below 5% and less than 20 points depressed on the 1% level on pattern deviation, plus no points in the central 5^0^ with a sensitivity of 0 dB and only one hemifield with a point with sensitivity of <15 dB within 5^0^ of fixation). Severe eyes (with MD ≤ −12 dB, < 50% of the points are depressed below the 5% and less than 20 points depressed on the 1% level on pattern deviation, at least one point in the central 5^0^ had a sensitivity of 0 dB and two hemifields had a point with sensitivity of <15 dB within 5^0^ of fixation) disease [32].

The inclusion criteria were the following: ametropia ≤ 0.5 dpt, an open anterior chamber angle (not less than 30°, See Study Examinations below) and no ocular pathology other than glaucoma.

The exclusion criteria included the following: large refractive errors (outside of ±6.00 dpt sphere or 2.00 dpt cylinder), pupil diameter < 3 mm, systemic administration of beta-blockers and calcium-channel blockers, concomitant ocular disease (except for early cataract), chronic autoimmune diseases, diabetes mellitus, Parkinson disease, Alzheimer disease, or dementia, history of stroke, acute circulatory disorders, including ocular arterial or venous obstruction (branch or central occlusion) in past medical history, and any concomitant disease involving the administration of steroid drugs. All systemic conditions associated with venous congestion (e.g. heart failure) were also considered as the exclusion criteria. Subjects treated with any substances for neuroenhancement, like nicergoline, citicoline, epigallocatechin-gallate or coenzyme q10, were excluded because these medicines may influence the retinal ganglion cell function [33]. Subjects were instructed to avoid caffeine intake, smoking, and exercise for 5 hours prior to the study visit.

If both eyes of a subject were eligible, one eye was randomly chosen.

Subjects who previously used antiglaucoma drops, were asked to discontinue the drug for a period of 21 days (drug washout period), while other subjects were newly diagnosed glaucoma cases. The medical histories of all subjects were carefully obtained with special attention directed toward the signs of primary or secondary cardiovascular dysregulation (migraine, vasospasm, neurocirculatory dystonia) [34].

All subjects underwent Doppler ultrasound scanning to exclude pathology of the brachiocephalic vessels.

The healthy participants were recruited from the persons accompanying the subjects and had IOP of less than 21 mmHg for both eyes, a normal Humphrey Swedish Interactive Threshold Algorithm (SITA) 24–2 standard visual field with MD, and pattern standard deviation (PSD) within 95% limits of the normal reference. They also had a glaucoma hemifield test within 97% limits, a central corneal thickness ≥500 μm, a normal-appearing ONH, a normal nerve fiber layer, an open anterior chamber angle as observed by gonioscopy, and no history of chronic ocular or systemic corticosteroid use. The age and race distribution of the controls matched that of the glaucoma subjects.

### Study examinations

All participants had complete ophthalmologic examinations including best corrected acuity, slit lamp examination, intraocular pressure (IOP) measurement using analyzer of biomechanical properties of the eye (Ocular Response Analyzer, ORA, Reichert Ophthalmic Instruments Inc., Depew, NY, USA), gonioscopy, anterior chamber angle measurement (Visante OCT, Carl Zeiss, Germany), pachymetry (SP-100, Tomey, GmbH, Erlangen, Germany), dilated fundus biomicroscopy using a 78-diopter lens, stereoscopic optic disc photography, and standard automated perimetry (SAP) using a Humphrey Field Analyzer (HFA, Carl Zeiss Meditec Inc., Dublin, CA, USA) with the 24–2 SITA. Only reliable SAP results, which were defined as false-negative and false-positive responses <33% and fixation losses <20%, were eligible for the study. Glaucomatous visual field defects were determined as having a cluster of 3 or more non-edge points with p < 0.05 and at least 1 point with p < 0.01 in the pattern deviation probability plot; PSD of less than 5%; or glaucoma hemifield test results outside normal limits. Both glaucoma and normal participants underwent SAP at least twice before this study.

Mean ocular perfusion pressure (MOPP) was calculated from IOP and arterial blood pressure (BP) measurements immediately before the OCT scanning and investigation of retrobulbar blood flow, after a 10-minute resting period in the sitting position. Systemic BP was measured using the Riva Rocci technique. MOPP was calculated using the formula: MOPP = (2/3 diastolic BP + 1/3 systolic BP) × 2/ 3 –IOP.

### OCT image acquisition and processing

All subjects also underwent optic disc area measurement on Avanti SD-OCT (Optovue, Inc., Fremont, CA, USA) using the traditional ONH scan. This scan consists of 12 radial scans 3.4 mm in length and 6 concentric ring scans ranging from 2.5 to 4.0 mm in diameter, all centered on the optic disc. The retinal pigment epithelium (RPE) tips are automatically detected by the software and are joined to delineate the optic disc margin and to calculate the disc area. All the examinations for a particular subject were performed on the same day. OCT was performed in the macula area as well. The tracking mode was used.

The GCC scan mode measured macular inner retinal layer thickness from the internal limiting membrane (ILM) to the inner nuclear layer–Avg. GCC. The GCC scan was centered on the fovea and covered a square grid on the central macula of 7.0×7.0 mm. The GCC thickness was determined with the GCC scanning protocol, which consists of 15 vertical line scans covering a 7.0×7.0 mm region centered about 1 mm temporal to the fovea. The GCC scanning protocol also included a central horizontal line scan for registration of vertical scans and fovea center searching. The characteristics of GCC (global loss volume–GLV, focal loss volume–FLV) were also measured.

The RNFL measurements in each area were automatically obtained and calculated using the retinal map protocol in the Avanti software. The full RNFL thickness was defined by the algorithm as the distance between the ILM and the middle of the RPE. The inner retinal layer thickness was defined by the algorithm as the distance between the ILM and the outer boundary of the inner plexiform layer (IPL). The average and regional information (superior hemisphere and inferior hemisphere) was obtained directly from this system.

The choroid thickness (CT) was examined in the tracking mode (Retina Cross Line protocol) as has been described previously [4].

### OCTA imaging of the optic disc, peripapillary region, and macula

OCTA scans were collected using the spectral-domain system (Avanti, Optovue Inc., Fremont, CA, USA): AngioVue OCTA software revision 2016.1.0.26.

**Fig 1:**
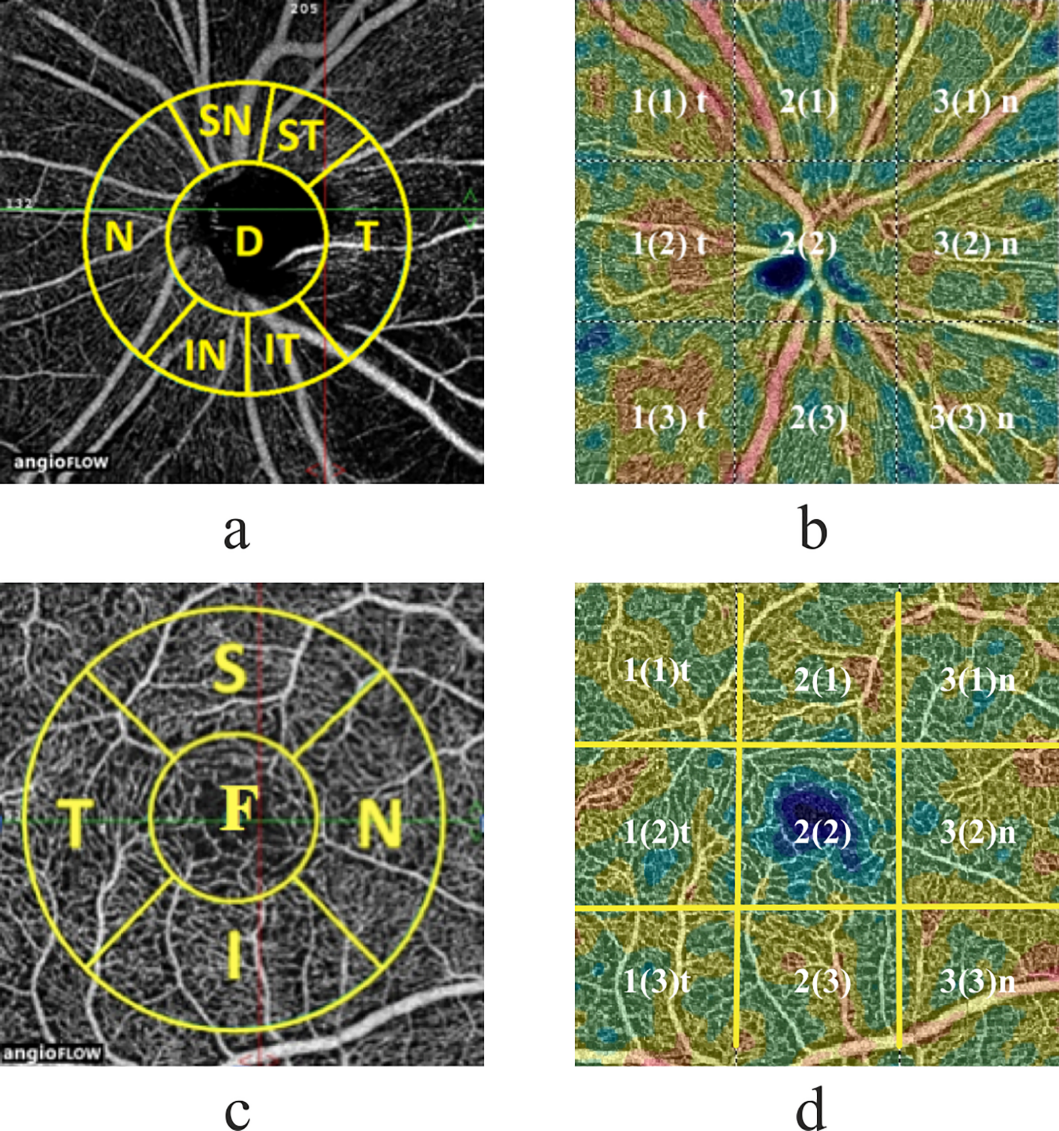
**Optic disc, peripapillary retina (a-b) and macular area (c-d) studied during OCTA.** (a) ONH and circumpapillary VD map measurement region defined: D–ONH (Inside Disc), SN–superonasal, ST–superotemporal, N–nasal T–temporal, IN–inferornasal, IT–inferotemporal. Peripapillary area: SN + ST + T + IT + IN + N. wiVD Disc: D + Peripapillary area. (c) Fovea (F) and circumparafovea VD map measurement region defined: S–superior, N–nasal, I–inferior sector, T–temporal. wiVD Macula: F+ circumparafovea; (b) and (d) VD color-coded maps of: ONH and circumpapillary area (b) and fovea and circumparafovea (d) divided into quadrants according to grid-based protocol; the figure without brackets specifies the column number, the figure in brackets–the row number, t–temporal, n–nasal.

The optic disc scan covers an area of 4.5×4.5 mm. The software automatically fits an ellipse to the optic disc margin (as detected by software) and calculates the average VD within the ONH (referred to as “inside disc” VD). The peripapillary region of 750-μm-wide elliptical annulus extending from the optic disc boundary is divided into 6 sectors based on the Garway-Heath map [35], and the peripapillary VDs are calculated in each sector (nasal, inferonasal, inferotemporal, superotemporal, superonasal, and temporal), as represented in Fig 1. Whole image vessel density (wiVD Disc), VD inside disc and peripapillary VDs were evaluated. Vessel density (VD, %) is defined as the proportion of the area occupied by vessels (white pixels) out of the whole area of the measurement sector. The peripapillary VD were analyzed in the radial peripapillary capillary (RPC) slab. The RPC slab extends from ILM to the posterior boundary of RNFL.

Macular scans cover a 6.0×6.0 mm area centered on fovea. Two vascular plexuses were studied in macula: a *superficial* plexus located in a retinal slab with the upper boundary 3 μm below the surface of ILM and lower boundary 15 μm below IPL, and a *deep* plexus located in the retinal slab with boundaries of 15 μm to 70 μm below IPL. Measurements were performed in the fovea (1mm diameter central zone) and parafovea (1-3mm diameter ring). The parafoveal region is divided into 4 sectors of 90 degrees each (nasal, inferior, superior, and temporal sectors). In addition, average VD for the whole image of the macula scan (wiVD macula) was evaluated. (Fig 1).

Image quality was assessed for all OCTA scans.

The retinal thickness measurements in each area were automatically obtained using the retinal map protocol in the Avanti software. The parafoveal thickness was measured within a circular annulus centered on the fovea using an OCT angiogram.

Only the images with optimal image quality (signal strength index >50), no motion artifacts, vitreous floaters, or other artifacts were selected.

### Electrophysiological studies

The visual evoked potentials (VEPs) and pattern electroretinograms (PERGs) were performed on electrophysiological system Tomey EP-1000 according the guidelines of International Society of clinical electrophysiology of vision [36,37].

VEPs are visually evoked electrophysiological signals extracted from the electroencephalographic activity in the visual cortex recorded from the overlying scalp as visual cortex is activated primarily by the central visual field, VEPs depend on functional integrity of central vision at all levels of the visual pathway including the eye, retina, the optic nerve, optic radiations and the occipital cortex [36].

PERGs are retinal responses evoked by a contrast-reversing pattern, usually a black and white checkerboard, which provides information on macular and retinal ganglion cell function [37].

Flash visual evoked potentials (FVEPs) were recorded in response to light-emitting diode white flashes of 2.0 cd*sec/m^2^ intensity given with frequency of 1 Hz. The viewing distance was 300 mm. The active recording electrode for VEPs was positioned on the Oz point of the scalp and the reference electrode on the Cz point. The ground electrode was on Fz. Ag/agcl polarized surface disk electrodes were used. For the FVEP latency and amplitude of peaks N2 and P2 were measured. For PVEP analysis, the N80, P100 and N135 waves latencies and amplitudes were assessed.

Pattern reversal VEPs (PVEPs) and transient PERGs (tPERGs) were recorded to a black and white reversing in counterphase checkerboard pattern with 98% contrast. Average luminance level was 50 cd/m^2^. A central fixation spot on cathode ray tube (CRT) monitor was used. For both the PVEP and PERG examinations the monitor was viewed at a distance of 1 m, at which a 15-degree stimulus field was achieved. Subjects were wearing appropriate spectacle correction for this distance. The check sizes of 1° and 0.3° were used. HK-loops-Ag fibers were used as active electrodes and Ag/agcl polarized surface disk electrodes—for reference and ground electrode. The analysis period (sweep time) for the tPERGs and steady-state (ssPERGs) was 200 ms. The first 30 sweeps of each response were rejected to allow steady-state conditions [38]. For tPERGs, the reversal rate was 4 reversals/s (2Hz) and for ssPERGs—16 reversals/s (8 Hz) [37]. In tPERGs, the amplitude and latency N35, P50 and N95 waves were analyzed. In ssPERGs, the measurement of P1 amplitude and phase shift (relative to the stimulus) of the response at the reversal rate (the second harmonic) using Fourier analysis was performed. To exclude the presence of maculopathy, macular (focal) electroretinograms were performed with normal results obtained.

The signal was band-pass filtered (1–100 Hz) and amplified (100,000 fold) for all electrophysiological examinations. Sweeps contaminated by eye blinks or gross eye movements were automatically rejected over a threshold voltage of 25 μV. A minimum of 150 artifact-free sweeps were recorded and averaged for reporting. As recommended by the International Society for Clinical Electrophysiology of Vision (ISCEV) guidelines, a double run was done for each wave to ensure reproducibility. Monocular stimulation was applied for VEPs. PERGs were obtained simultaneously in both eyes of each subject.

### Statistical data processing

We applied generalized Mann–Whitney–Wilcoxon rank sum test using Rosner-Glynn-Lee method. Parameters with p-value < 0.05 were considered statistically significant. As a parameter’s importance measure for distinguishing the groups, we used area under receiver operating characteristic curve (AUC). First, we examined correlations between the parameters separately in the groups of healthy control eyes, early and moderate to severe POAG. Parameters with very strong correlation were combined into clusters. Afterwards, for differentiating the early POAG from the control eyes and between the POAG stages, from each cluster one parameter with the largest value of AUC was chosen.

Since several parameters (GCC, GLV, systolic and mean perfusion pressure, CT) depended on the anterior-posterior axis and the age of the subjects, we carried out an adjustment for these parameters on the basis of the linear regression model.

To examine the relation between the characteristics, we used the Spearman correlation coefficient. Its significance was measured by p-value of slope coefficient in mixed model. Statistical analysis was performed using the SPSS version 21, MASS and nlme packages in R.

## Results

The general characteristics of the POAG subjects and control subjects are summarized in Table 1.

**Table 1:** Characteristics of normal subjects and subjects with glaucoma.

Parameter	Healthy eyes (n: 35)	AUC* p*	Early POAG (n: 48)	AUC** p**	Moderate to severe POAG (n: 42)
Age, years	62.4 (5.5)		63.7 (7.2)		65.1 (6.0)
Systolic BP, mm Hg	124.5 (4.6)	0.707 (0.09) 0.05	136.2 (17.9)		131.4 (14.1)
Diastolic BP, mm Hg	81.4 (6.9)		84.4 (11.2)		82.8 (8.4)
Corneal compensated IOP, mm Hg	13.5 (3.1)	**0.870 (0.07) 0.001**	19.3 (4.6)		19.8 (6.3)
MOPP, mm Hg	50.4 (2.4)		47.8 (10.0)		46.2 (7.9)
MD, dB	0.0 (0.8)	**0.800 (0.07) 0.001**	–2.1 (3.4)	**0.924 (0.04) <0.001**	–11.8 (6.1)
PSD, dB	1.4 (0.2)	0.745 (0.07) 0.008	2.2 (1.6)	**0.951 (0.03) <0.001**	9.7 (3.7)
RNFL, μm	98.9 (8.9)	0.720 (0.09) 0.012	91.5 (9.7)	**0.881 (0.05) <0.001**	70.0 (14.3)
GCC, μm	97.2 (10.9)	0.739 (0.09) 0.016	88.2 (10.1)	**0.886 (0.05) <0.001**	71.3 (11.4)
FLV, %	0.8 (1.3)		1.1 (1.7)	**0.923 (0.03) <0.001**	9.4 (4.2)
GLV, %	3.3 (4.1)	0.714 (0.09) 0.025	8.1 (9.7)	**0.904 (0.04) <0.001**	25.1 (10.5)
Axial length, mm	23.5 (0.8)		23.4 (1.4)		23.3 (0.5)
Corneal thickness, μm	535 (25.6)	0.823	543.6 (30.2)	0.254	536.7 (29.3) 0.864
Foveal CT, μm	312.8 (88.5)		279.9 (105.2)		272.6 (82.1)
Peripapillary CT, μm	181.4 (51.3)		179.3 (93.3)		161.9 (64.9)

The table shows the mean values and standard deviation (in parentheses), clustered Area under ROC-curve (AUC*) and clustered Wilcoxon rank sum test using Rosner-Glynn-Lee method (p*) between the healthy eyes (control group) and early POAG, p** between the POAG groups. Here and in other tables the AUCs in the corresponding cells are not given in the absence of a significant (at 0.05 level) difference between groups. All abbreviations are given in the list of abbreviations.

There were no significant differences in age, sex, refractive error, and ocular perfusion pressure (OPP) between the studied groups as well as in IOP between the early glaucoma and moderate to severe POAG. The IOP in early glaucoma was significantly higher compared to normal subjects. Significant differences were found between the studied groups for MD, PSD and all other parameters, except for the corneal, foveal and peripapillary choroidal thickness.

**Fig 2:**
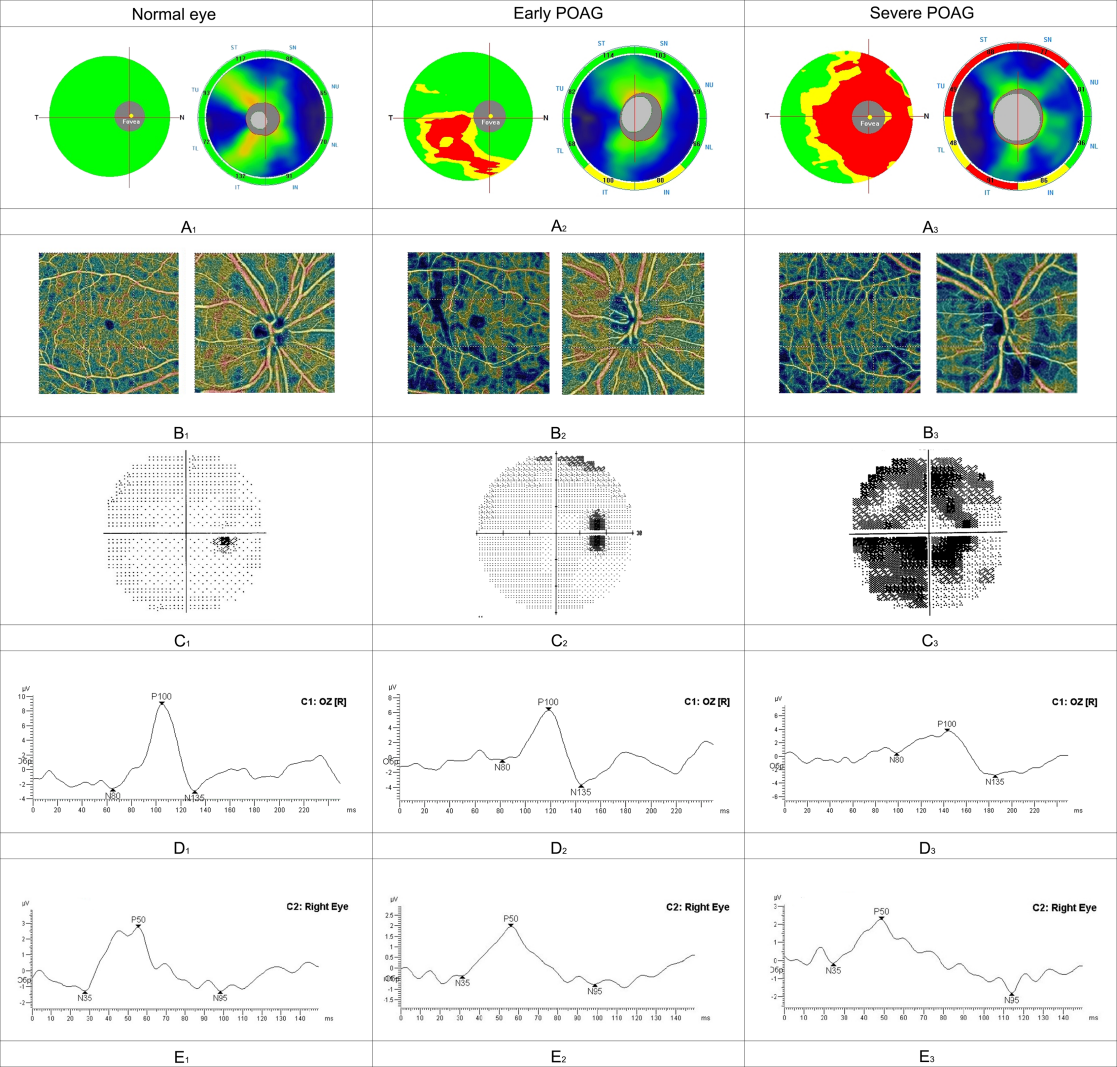
Clinical examples of the normal controls, early glaucoma, and severe glaucoma. GCC map and RNFL thickness map **(Fig 2A)**, SAP visual field results showing corresponding visual field defects **(Fig 2C)**, PVEP-protocols **(Fig 2D)**, PERG-protocols **(Fig 2E)**. Fig **2B** show a stepwise decrease of vessel density both in the circumpapillary VD map and Fovea and circumparafovea VD map (wiVD Disc is reduced from 54.25% (normal eye) to 52.26% (early glaucoma) to 42.17% (severe glaucoma); wiVD Macula Superficial is reduced from 52.56% (normal eye) to 41.95% (early glaucoma) to 41.29% (severe glaucoma). Fig **2D** show a stepwise decrease of the amplitude and prolonged latency of P100 component of PVEP and Fig **2E** show a decrease of the amplitude and prolonged latency of N95 component of PERG in glaucoma eyes compared to normal eye.

The results of OCTA in the ONH, peripapillary and macular areas in glaucomatous and normal participants are shown in Table 2. Compared to the healthy controls, the early glaucoma group had capillary network dropout inside disc, fovea, peripapillary and parafoveal areas, especially in the inferior macular region. In general, the results demonstrated more significant differences between the normal eyes and early glaucoma in the macula (Fig 2). Meanwhile, comparing the POAG stages, a significant difference was revealed mostly between the VD inside disc and in peripapillary area (Table 2, Fig 2).

**Table 2:** Vessel density measurements of study participants.

Variables	Normal eyes	AUC* p*	Early POAG	AUC* p**	Moderate to severe POAG
Disc scan					
wiVD Disc (disc scan)	55.8 (2.2)	0.740 (0.07) 0.016	52.9 (4.1)	**0.895 (0.04) <0.001**	43.1(6.2)
Inside Disc VD	50.2 (4.3)	0.756 (0.08) 0.009	45.3 (6.5)	**0.841 (0.05) <0.001**	37.7 (5.6)
Peripapillary VD	58.0 (2.8)		56.9 (4.9)	**0.879 (0.05) <0.001**	46.3 (7.4)
Nasal VD	56.9 (4.1)		54.8 (5.7)	**0.861 (0.05) <0.001**	46.5 (6.4)
Inferonasal VD	55.8 (4.0)	0.683 (0.08) 0.008	57.6 (7.4)	**0.878 (0.05) <0.001**	45.0 (8.2)
Inferotemporal VD	60.5 (3.5)		59.8 (4.4)	**0.936 (0.04) <0.001**	44.4 (8.9)
Superotemporal VD	60.4 (3.6)		59.0 (4.8)	**0.807 (0.08) <0.001**	49.2 (10.0)
Superonasal VD	55.6 (4.3)		54.7 (7.3)	**0.778 (0.08) <0.001**	44.8 (10.3)
Temporal VD	59.4 (4.2)		58.0 (6.9)	**0.787 (0.07) <0.001**	47.0 (11.8)
Macula scan					
wiVD Macula Superficial	50.7 (3.0)	**0.800 (0.06) 0.001**	45.9 (5.0)	0.679 (0.08) 0.017	42.2 (6.0)
Foveal VD superficial	36.4 (5.7)	0.689 (0.09) 0.02	32.1 (6.4)		30.8 (6.4)
Parafoveal VD superficial	51.9 (5.2)	0.750 (0.08) 0.006	47.0 (5.5)		46.3 (5.2)
Temporal	52.4 (5.4)	0.742 (0.08) 0.007	47.2 (5.9)		46.1 (6.0)
Superior	53.2 (5.2)	0.793 (0.07) 0.003	47.3 (5.3)		47.4 (5.4)
Nasal	51.2 (5.9)	0.704 (0.09) 0.02	46.8 (6.4)		46.3 (6.2)
Inferior	50.8 (6.1)	**0.687 (0.09) 0.001**	43.5 (6.5)		45.3 (5.8)
wiVD Macula Deep	57.3 (4.1)	0.764 (0.07) 0.005	52.2 (6.9)	0.697 (0.07) 0.01	46.6 (8.5)
Foveal VD Deep	35.4 (7.6)		32.5 (7.9)	0.720 (0.07) 0.004	26.3 (8.6)
Parafoveal VD Deep	60.2 (3.7)	0.738 (0.07) 0.016	56.3 (5.4)	0.711 (0.08) 0.006	51.5 (6.9)
Temporal VD	60.0 (4.1)	0.696 (0.08) 0.046	55.8 (6.7)	**0.771 (0.07) 0.001**	49.2 (7.3)
Superior VD	62.1 (4.0)	0.788 (0.06) 0.002	57.0 (5.1)	0.625 (0.08) 0.05	54.1 (6.7)
Nasal VD	59.6 (4.9)	0.683 (0.09) 0.037	56.3 (5.9)	0.708 (0.09) 0.006	50.7 (8.7)
Inferior VD	59.3 (4.6)		56.0 (6.6)	0.661 (0.07) 0.025	52.0 (7.3)

The table shows the mean values and standard deviation (in parentheses), clustered Area under ROC-curve (AUC*) and clustered Wilcoxon rank sum test using Rosner-Glynn-Lee method (p*) between the healthy eyes (control group) and early POAG, p** between the POAG groups.

VD–vessel density (%) is the ratio of the area of the vessels in the test spot to the area of the test.

wiVD Disc–whole en face image vessel density (disc scan), wiVD Macula–whole en face image vessel density (macula scan).

When analyzing the results of electrophysiological studies (EPS), we found that subjects with early glaucoma differed from the healthy controls in the amplitude of P50, N95 components of tPERGs and ssPERGs. The amplitude of the P100 component of PVEP was significantly different in all studied groups. The results of the electrophysiological testing are summarized in Table 3.

**Table 3:** Electrophysiological parameters in the studied groups.

Parameter	Normal Subjects	AUC* p*	Early POAG	AUC** p**	Moderate to severe POAG
Amplitude of P100 component of PVEP 0,3°, μv	15.8 (3.8)		11.9 (6.4)	0.713 (0.09) 0.01	7.8 (4.6)
Amplitude of P100 component of PVEP 1°, μv	16.3 (3.1)	0.835 (0.08) 0.01	11.2 (6.1)	0.733 (0.09) 0.004	7.1 (3.5)
Amplitude of P50 component of tPERG, 1°, μv	5.7 (1.5)	0.930 (0.05) 0.002	2.8 (1.6)		2.7 (1.7)
Amplitude of N95 component of tPERG, 1°, μv	7.0 (1.8)	0.893 (0.06) 0.002	3.7 (1.8)		3.4 (2.2)
Amplitude of P1component of ssPERG, μv	3.0 (0.6)	0.915 (0.05) 0.003	1.7 (0.7)		1.5 (0.8)

The table shows the mean values and standard deviation (in parentheses), clustered Area under ROC-curve (AUC*) and clustered Wilcoxon rank sum test using Rosner-Glynn-Lee method (p*) between the healthy eyes (control group) and early POAG, p** between the POAG groups. All abbreviations are given in the List of abbreviations.

**Fig 3:**
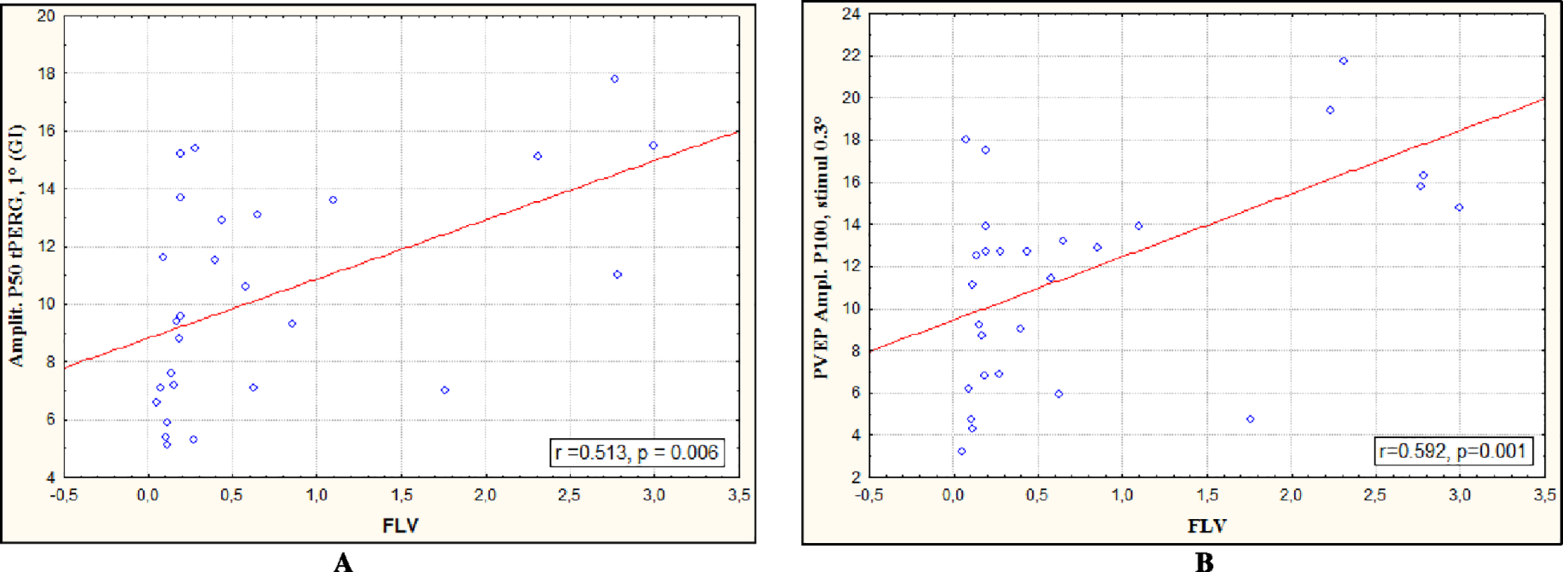
Correlations between the electrophysiological data and Focal Loss Volume of GCC: (a) amplitude P50 tPERG, (b) amplitude P100 PVEP in early POAG.

Strong correlations were found between the amplitude P50 tPERG and focal loss volume (FLV) of GCC as well as between the amplitude P100 PVEP and FLV in early glaucoma **(Fig 3).**

On the other hand, the strong correspondence was revealed between the amplitude P100 PVEP and the vessel density in ONH and peripaillary retina in the advanced glaucoma group (Table 4).

**Table 4:** Correlations between the electrophysiological data and OCTA parameters in moderate to severe POAG.

Variables	Amplit. P50 tPERG, 1°	PVEP Ampl. P100, stimul 0.3°	PVEP Ampl. P100, stimul 1°
wiVD Disc		r = 0.714; p = 0.02	r = 0.521; p = 0.067
Avg. peripapillary VD		r = 0.63; p = 0.013	
Macula Grid-Based 2 (3)	r = 0.689; p = 0.03		
Disc Grid-Based 1(1)t		r = 0.564; p = 0.028	r = 0.547; p = 0.053
Disc Grid-Based 1(3)t		r = 0.584; p = 0.052	r = 0.559; p = 0.054

r–Spearman correlation coefficient. All abbreviations are given in the List of abbreviations.

Peripapillary superotemporal 1(1)t and inferotemporal 1(3)t areas are marked according to Fig 1B and macular inferior area is marked 2(3) according to Fig 1D.

**Fig 4:**
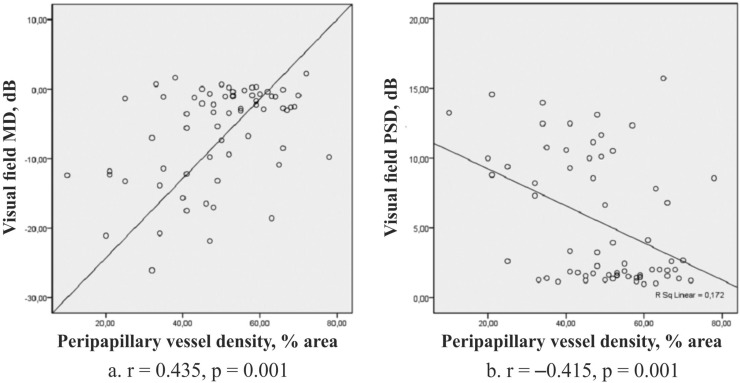
Correlations between the average peripapillary VD and visual field indexes: (a) mean deviation (MD), (b) pattern standard deviation (PSD) in early glaucoma.

**Fig 5:**
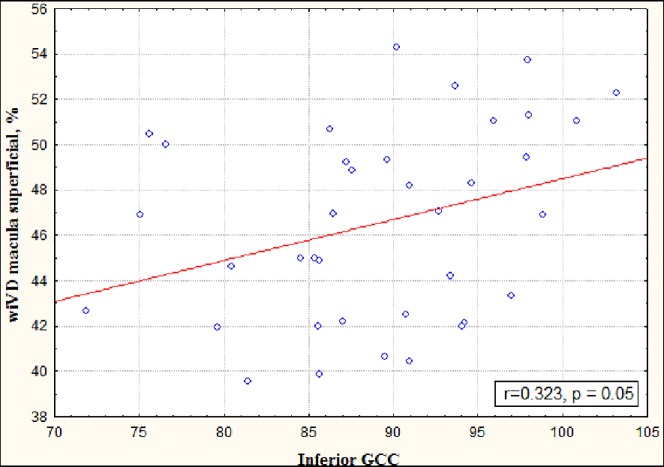
Correlation between the vessel density in the superficial retinal plexus of macula and the GCC thickness in the inferior hemisphere.

The parameters of OCTA strongly corresponded to the visual field indexes (Fig 4) and to the GCC thickness in inferior hemisphere (Fig 5).

In early glaucoma the negative correspondence was found between the peripapillary choroidal thickness and whole en face vessel density in Disc scan as well as in the superficial parafovea area. The foveal choroidal thickness negatively corresponded to the vessel density in the macular superficial plexus (Table 5).

**Table 5:** Correlations between the OCTA data and the choroidal thickness in early glaucoma.

Variables	wiVD Disc	VD Superficial Parafovea	wiVD macula superficial	Grid-Based VD Superficial in inferior parafovea	OCT thickness ILM–IPL inferior hemisphere
CT peripapillary	**r = –0.445** **p = 0.01**	r = –0.102 p = 0.43	r = –0.150 p = 0.132	r = –0.22 p = 0.045	r = –0.124 p = 0.23
CT in fovea	r = 0.05 p = 0.546	**r = –0.642** **p = 0.009**	**r = –0.651** **p = 0.005**	r = –0.245 p = 0.29	r = –0.344 p = 0.65

All significant correlations are given in bold. All abbreviations are given in the list of abbreviations.

Based on the obtained results, the parameters with the highest diagnostic accuracy were detected for distinguishing the studied groups of glaucoma subjects. For this purpose, parameters with very strong correlation were combined into clusters. Afterwards, for differentiating the early POAG from the control eyes and between the POAG stages, from each cluster one parameter with the largest value of AUC was chosen. As a result, several parameters were selected for early glaucoma detection, that are represented in Table 6. The parameters with the highest AUC for distinguishing between the early POAG and the moderate to severe POAG are listed in Table 7. According to this data, the parameters of PERG and VD in fovea/parafovea had the best diagnostic accuracy for early glaucoma diagnostics, while the inferotemporal peripapillary VD and focal loss volume of GCC had the highest diagnostic value for discriminating the early glaucoma from the moderate to severe glaucoma (Table 7).

**Table 6:** Diagnostic ability of studied clinical parameters in differentiating early POAG from healthy eyes.

Parameter	Normal Subjects	AUC p	Early POAG
Amplitude of P50 component of tPERG, 1°	5.7 (1.5)	0.930 (0.05) 0.002	2.8 (1.6)
Amplitude of P1 component of ssPERG	3.0 (0.6)	0.915 (0.05) 0.003	1.7 (0.7)
Amplitude of P100 component of PVEP, 1°	16.3 (3.1)	0.835 (0.08) 0.013	11.2 (6.1)
wiVD macula Superficial	50.7 (3.0)	0.800 (0.06) 0.001	45.9 (5.0)
wiVD Disc	55.8 (2.2)	0.740 (0.07) 0.016	52.9 (4.1)
GCC	97.2 (10.9)	0.739 (0.09) 0.016	88.2 (10.1)

AUC–area under receiver operating characteristic curve.

All abbreviations are given in the list of abbreviations.

**Table 7:** Diagnostic ability of studied clinical parameters in differentiating early glaucoma from moderate to severe glaucoma.

Parameter	Early POAG	AUC p	Moderate to severe POAG
Inferotemporal Peripapillary VD	59.8 (4.4)	**0.936 (0.04)** **<0.001**	44.4 (8.9)
FLV	1.1 (1.7)	**0.923 (0.03)** **<0.001**	9.4 (4.2)

AUC–area under receiver operating characteristic curve.

All abbreviations are given in the list of abbreviations.

## Discussion

The present study compares the diagnostic ability of the vascular, structural and functional parameters in differentiation between the normal eyes, early glaucoma and moderate to severe glaucoma. We have revealed that the results of the electrophysiological testing along with the retinal microcirculation measured by OCTA demonstrated superiority over the structural variables in early glaucoma detection.

PERGs have been reported to have the highest specificity and sensitivity in glaucoma among all functional diagnostics methods [24–26,29,34–39].

Our results show that the N95 wave PERG amplitude was significantly lower in early glaucoma compared to the normal eyes and concur with the previous findings demonstrating reduction in the PERG amplitude in glaucoma without significant alteration in latency [25–28]. The P50 component of tPERG is used to assess functional state of the middle layers of the macular area, the N95 component–of the RGCs. It is worth noting that the PERG changes in glaucoma can be observed before the abnormalities in the ONH and peripapillary retina appear. PERGs allow detecting transformation from ocular hypertension into glaucoma several years before early glaucoma occurs, and shows a high sensitivity (up to 75%) [40,41]. According to the literature, ssPERGs are more sensitive measures of ganglion cells dysfunction than all other functional tests [42–43]. It has been revealed that the alterations of the ssPERG amplitude indicated adaptive changes reflecting the neuronal activity of the inner retina. Thus, the ssPERG amplitude represents functional parameters that are different from the parameters of standard PERG and are more informative in early glaucoma detection [44]. This is confirmed by our data as the amplitude of P1 component of ssPERG was selected for early glaucoma detection (Table 6).

Having compared the diagnostic value of the structural indicators and PERG data, Bowd et al. concluded that PERGs appeared to be a more sensitive method of glaucoma diagnostics than OCT, and the functional changes recorded by PERGs were ahead of structural ones, which today can most accurately be evaluated only by spectral OCT (SD-OCT). According to the literature, PERGs allow proper identification of the eyes with glaucoma risk before the onset of common symptoms [24,44–45]. For this reason, we included PERGs in our study and confirmed their advantage over structural measurement by SD-OCT in early glaucoma detection (Table 6). Moreover, the high correspondence between amplitude P50 tPERG and focal loss volume of GCC reflects the dysfunction of ganglion cells in early glaucoma. This is confirmed by a strong association between the amplitude P100 of PVEP and FLV (Fig 3).

According to literature, VEPs for pattern reversal of 1° and 0.3° are also very sensitive for glaucoma damage. Furthermore, PVEPs may reflect the function of RGCs objectively and sensitively. It was emphasized in literature that P100 can be used as a measure of early glaucomatous damage before the RGCs death [29,46].

Our results have revealed that the compared groups differed mostly in the amplitude of P100 PVEP for the small and large pattern. These findings are consistent with those of other authors [30,31]. The literature on VEP in glaucoma is scarce; moreover, much less attention is paid to association of VEP parameters with indicators of ocular blood flow. According to Mokbel and Ghanem [12], the reduction of blood flow in the central retinal artery and ophthalmic artery is an important indicator of a violation of the blood supply and function of the optic nerve in glaucoma, which is reflected as reduced P100 amplitude of PVEP and coincides with our previous findings [7].

The data obtained on the evaluation of electrophysiological testing in our study confirms the assumption of the RGCs dysfunction in early glaucoma. It is known that the integrity of the innermost retinal layers in humans is required to obtain a normal PERG response [29,41,47–49]. PERGs may play a clinical role as direct and objective indexes of the function of RGCs and their fibers. The decrease in P50 to N95 amplitude has been reported as an indicator of a dysfunction of RGCs and their fibers [24,37,40–44,47]. Parisi et al. [29] established that the complex PERG/VEP records identified a large percentage of ocular hypertension eyes with impairment of the innermost retinal layers, notwithstanding normal optic disc morphology and normal HFA-indices. The results of this group demonstrated that in POAG eyes, PERG P50 to N95 amplitude and VEP P100 implicit time showed the highest sensitivity/specificity for the detection of a visual dysfunction. In both POAG and ocular hypertension eyes, a delay in PERG P50 implicit time and a reduction in P50 to N95 amplitude were described. However, the delay in the P50 component observed in these eyes cannot be ascribed exclusively to a pure RGC dysfunction because of the contribution of preganglionic elements has been suggested in the genesis of the P50 component [29,47]. This agrees with the experimental studies in animal models of glaucoma where RGC degeneration and pharmacological blockage is followed by a reduction in PERG amplitude involving the P50 to N95 complex [48] and proved in further studies on the origins of PERGs showing that N95 reflects spiking activity of ganglion cells and P50 reflects non-spiking activity as well [49].

At the same time, the data of Parisi et al [29] supports the hypothesis that RGC dysfunctions preceding their death may be reflected in PERG results, whereas HFA sensitivity is still unaltered.

To better understand the nature of dysfunction of GCC and their axons detected by EPS, we used OCTA to study the retinal microvascularity and evaluated VD both in macula and in ONH and peripapillary retina. Our results have revealed the strong correspondence between the amplitude P100 PVEP and the vessel density in ONH and peripapillary retina on the one hand and the correlation between the vessel density in the superficial macular plexus and the GCC thickness in inferior hemisphere on the other hand. On the basis of this data one may assume that ganglion cells dysfunction may be related to the reduction of retinal blood supply. Our results agree with findings of Xu, who showed that the macular capillary vessel area density strongly correlated with inferior hemimacular structural damage [50]. This finding indicates that the inferior hemimacular retinal structure is susceptible to a decrease in the retinal capillary vessel area density in glaucomatous eyes. Our previous study revealed that the blood flow parameters in ophthalmic artery, central retinal artery and short posterior ciliary arteries in early glaucoma were below normal and significantly correlated with the retinal thickness in the inferior hemisphere [51]. According to our previous results, the retinal thickness in the subjects with early glaucoma differed significantly from the retinal thickness in the healthy subjects only in the inferior macula hemisphere [18].

Different authors found a significant decrease in the ONH VD in glaucoma subjects as compared with healthy subjects. Wang et al. reported reduced blood flow index in the entire optic disc and inferotemporal segment of the optic disc [15]. Chichara et al. revealed the priority of detection of the superficial retina peripapillary VD for differentiating the glaucoma and ocular hypertension from the normal eyes [52]. Liu et al. revealed a significant decrease in the peripapillary VD in subjects with glaucoma as compared with healthy subjects of similar age [16]. In the authors’ opinion, this parameter was of high diagnostic value for early detection of glaucoma. Some other studies have reported that quantitative analysis of OCTA was able to differentiate glaucomatous eyes from normal eyes by means of assessing the entire peripapillary vasculature, from the ILM to the Bruch’s membrane [19]. According to Yarmohammadi et al., the decreased VD was significantly associated with the severity of visual field damage independent of the structural loss, and wiVD of the disc scan showed the best AUC in their study (AUC: 0.94) [20].

It is worth noting that in all these studies the authors concentrated on the OCTA parameters of the vessels supplying the ONH. Meanwhile, it has been emphasized in the literature, that the macula consumed more oxygen per weight than any other tissue and was likely susceptible to hypoxic and ischemic damage [53]. Considering that most RGCs resided in the inner retinal layers, completely supplied with oxygen and nutrients from the superficial retinal capillary plexus, it is clear that the inner retinal layer, especially the RGC, is extremely susceptible to damage from the dropout of retinal capillaries. In our previous study we reported on the significant reduction of VD in macula in early glaucoma compared to the normal eyes [18]. These findings were consistent with those of Xu’s study, which showed that eyes with early glaucoma had a lower macular capillary vessel area density, compared to healthy eyes, and microvasculature deficits were correlated with layer damage, and visual function defects [54]. Recently, Takusagawa et al. also demonstrated the early decrease of VD in the superficial macula plexus using the Project Resolved OCTA [54].

These results are at variance with some literature data. For example, Rao and coauthors reported that the parameters with the highest diagnostic abilities to differentiate the glaucoma eyes from the normal eyes were the wiVD of the disc scan (AUC of 0.90) and the inferotemporal sector peripapillary VD (AUC of 0.89) [55]. This may be owing to the more severe glaucoma in their cohort (median MD: –6.3 dB compared to –1.94 dB in our subjects with early glaucoma). Furthermore, in contrast to the design of our study, only the pretreatment IOP was assessed by Rao and coauthors. They revealed that the pretreatment IOP had a significant positive relation with the AUCs of the inside disc VD, but did not influence the AUCs of the peripapillary or the parafoveal measurements. Thus, according to Rao, the diagnostic ability of ONH VD increased in eyes with higher baseline IOP. This may imply that the vascular mechanisms contributing to the pathogenesis of glaucoma are not IOP-independent. On the other hand, the authors assumed that the VD decrease in the macular and the peripapillary retinal vessels in glaucoma might be independent of the IOP levels at which the glaucoma develops. According to Hollo, the high IOP level may comprise the retina and its small vessels in the RNFL, thus it may reduce peripapillary capillary perfusion [56]. In contrast to this, Scripsema et al didn’t obtain any link between the vessel density and IOP [57]. We also failed to find a correlation between the IOP and the OCTA parameters in any of the studied regions–neither in the ONH and peripapillary retina nor in the macula, though all the subjects stopped using hypotensive eye drops three weeks before the examination. The same results were obtained in our previous study [51]. This suggests that VD decreases in the macula, and the peripapillary retina in glaucoma might be IOP-independent. More studies are required to understand which IOP level may damage the retinal microvascularity measured by OCTA.

In the present study, we revealed a decrease of the PERG amplitude and VD in the inner layers of the macular area in both vascular plexuses in early glaucoma. When comparing these markers with the wiVD measured over the entire image of the circumpapillary area and the optic disc, the importance of the macula parameters for early detection of the disease was greater (Table 6). In contrast to early glaucoma, the subjects with moderate to severe stage had a stronger deficit of capillaries density in the circumpapillary area, particularly in the inferotemporal sector (Table 7). VD in the macula had the highest diagnostic ability over those most important structural parameters that had so far been recognized as high-priority in the early diagnosis of glaucoma (RNFL thickness, GCC and its characteristics). In the present study we did not select RNFL as the parameter with a high diagnostic accuracy as its AUC (0.69, confidence interval (CI) 0.58–0.82) was below AUC for GCC thickness (0.74, CI 0.6–0.86), and both parameters strongly correlated with each other belonging to the same cluster of diagnostic markers in statistical analysis. Meanwhile, in many studies it has been reported that RNFL thickness in the inferior quadrant had the best performance of discriminating the early glaucoma from the normal eyes and that the inferior region of the optic disc was commonly affected [3, 58]. Furthermore, the authors assumed that the inferior macular region was also very susceptible to the glaucomatous damage.

Based on the combined data, we assume that the lack of retinal blood supply may lead to the reduction of visual function and structural loss in early glaucoma. Thus, our results concur with the findings of other authors on the inferior and temporal ONH vulnerability to early glaucomatous optic nerve atrophy [58,59] and support the assumption that retinal microvasculature abnormalities and structural damage occur more easily in the inferior areas of the retina.

When comparing the groups of glaucoma subjects, the vascular density in the inferotemporal peripapillary retina was the most important marker, which, along with the focal loss volume of GCC, allowed to differentiate early glaucoma from advanced one. According to Rao et al., compared the subjects with glaucoma (MD –6.5 dB) with healthy subjects, the diagnostic abilities of ONH, peripapillary retina and the macular VDs in POAG were significantly lower than peripapillary RNFL and macular GCC measurements [55]. We suppose that these findings are partly consistent with our data that has been discussed above. Herein, we emphasize that the diagnostic ability of the retinal VD, while comparing with structural changes, much depends on the stage of glaucoma: it may be more pronounced for the early glaucoma detection than for its monitoring.

One finding of this study, that remains unclear, is a negative correspondence between the peripapillary choroidal thickness and whole en face vessel density in disc scan as well as in the superficial parafovea area on the one hand, and between the foveal choroidal thickness the vessel density in the macular superficial plexus on the other hand. Previously we have found the increased choroidal thickness in pre-perimetric glaucoma than in healthy individuals [4]. Whether this relates to a compensatory mechanism of the choroid or is related to increased pressure in ocular veins is unclear. Indeed, factors that influence the choroidal thickness and its relations to the retinal microvasculatory are still to be determined.

According to the results of the present study, one can assume that trophic deterioration in the capillaries of the inner retina layers may be the reason of the involvement of the macula and inferotemporal peripapillary retina in the pathological process at the very beginning of the disease. This was confirmed by the fact that in early glaucoma the peripapillary VD had a link with perimetric indexes (Fig 4). We have recently revealed that at this stage of the disease the higher resistive index of ophthalmic artery was associated with less VD in foveal and parafoveal superficial plexus. Furthermore, in early glaucoma the wiVD Disc parameter negatively correlated with the end diastolic velocity in the temporal short posterior ciliary artery [51]. In the normal eyes there was an inverse correlation between the OPP and VD in the macula superficial plexus though other authors did not find the relation between the blood pressure readings and retinal VD [55]. We explain it with the autoregulation of ocular blood flow that is present in normal eyes and may be still undamaged at the very beginning of the disease. It has been previously shown that the reduction of retinal microcirculation may occur as an autoregulatory response during hyperoxia [60]. One may speculate that significant blood supply to the retina due to the higher blood flow in ophthalmic artery causes the constriction of small retinal capillaries. This leads to the reduction of the blood flow in the retinal vascular plexuses and some of their vessels cannot be detected by OCTA anymore.

The current study has several limitations that should be considered while interpreting the results. Only subjects with SAP abnormal visual fields were recruited, and thus, the current study cannot answer the question–which functional, structural or circulation parameter shows the highest diagnostic ability in preperimetric glaucoma. To investigate such a question, subjects should be recruited based on the criteria not including SAP results. However, SAP is the current standard method to verify the glaucoma diagnosis and to monitor time course of functional damage, and the purpose of the current study was to compare the diagnostic abilities of structural, functional and circulatory parameters in the diagnosis of early glaucoma and in its monitoring.

In this study we failed to demonstrate any link between the studied parameters in healthy eyes because of the limited data. Moreover, the mixed-effect models of statistical analysis have reduced the number of significant correlations between the parameters for glaucoma groups as well.

As our study is not longitudinal, it is impossible to assess the changes over time in each subject. Therefore, we cannot conclude whether the macular, ONH or peripapillary microvasculature changes cause or result from degenerative glaucomatous processes.

The current study was conducted in POAG subjects with a moderate elevated IOP. Indeed, their IOP after the eye drops washout period was rather low according to Table 1. However, all subjects had a documented history of IOP > 21 mm Hg. On the other hand, PERG responses may be affected by elevated IOP or by its lowering [61–65]. At the same time, some authors did not reveal any effect of medication use on the PERG amplitude [66] or postulate that it may occur only in early glaucoma [62] or mostly depending on IOP fluctuations [67].

Finally, it has been postulated that the PERG amplitude in treated subjects may not accurately represent the true disease state because induced IOP decreases may result in concomitant increases in amplitude [68]. We consider that this is a strong side of our study as we stopped the administration of anti-glaucoma medications in POAG subjects before the examination. We believe that this kind of treatment may affect both PERG results and VD measured by OCTA and give incorrect information on the role of OCTA and PERG in glaucoma diagnostics. Another advantage of our study is the correspondence revealed between the capillary retinal density and the function of GCC and their axons forming visual track, evaluated by EPS at different glaucoma stages. The application of the OCTA grid-based algorithm also supports the strength of this study allowing to assess focal correspondence among capillary network defects, structural damage and functional loss.

## Conclusions

Our results demonstrate the importance of measuring the microcirculation parameters in the macular area along with PERGs and PVEPs for the early detection of glaucoma. VD in the inferotemporal sector of the peripapillary retina and focal loss volume of GCC are important for monitoring of the disease. The including of OCTA, PERGs and PVEPs in glaucoma diagnostics may improve its early detection and monitoring.

## Abbreviations

AUC: area under the receiver operating characteristic curve
BP: blood pressure
CI: confidence interval
CT: choroidal thickness
EPS: electrophysiological studies
FLV: focal loss volume
GCC: ganglion cell complex
GLV: global loss volume
ILM: internal limiting membrane
IOP: intraocular pressure
IPL: inner plexiform layer
MD: mean deviation
MOPP: mean ocular perfusion pressure
OPP: ocular perfusion pressure
OCT: optical coherence tomography
OCTA: optical coherence tomography angiography
ONH: optic nerve head
PERG: pattern electroretinogram
POAG: primary open-angle glaucoma
PSD: pattern standard deviation
PVEP: pattern visual evoked potential
RGC: retinal ganglion cell
RNFL: retinal nerve fiber layer
RPC: radial peripapillary capillary
RPE: retinal pigment epithelium
SAP: standard automated perimetry
SSADA: split-spectrum amplitude-decorrelation angiography
SD-OCT: spectral-domain optical coherence tomography
SITA: Swedish Interactive Threshold Algorithm
ssPERG: steady-state PERG
tPERG: transient PERG
VD: vessel density
VEP: visual evoked potential
wiVD: whole en face image vessel density
